# Supplementary material for: Mechanism of One-Step Hydrothermally Synthesized Titanate Catalysts for Ozonation
Source: Molecules. 2022 Apr 22;27(9):2706. doi: 10.3390/molecules27092706 (PMC9103479; doi:10.3390/molecules27092706)

Supplementary Material

Mechanism of one-step hydrothermally synthesized titanate catalysts for ozonation

Geshan Zhang [1,*], Anhua Jiang [2], Xinwen Huang [2,*], Tian Yuan [1], Hanrui Wu [1], Lichun Li [1], Zongjian Liu [1]

1 College of Chemical Engineering, Zhejiang University of Technology, Hangzhou 310014, China.

2 College of Environment, Zhejiang University of Technology, Hangzhou 310014, China.

[*]Corresponding author Email: zhanggs@zjut.edu.cn

Tel: +86-0571-8832-0412

Figure S1 ln(C_0/C) versus t when applying synthesized titania catalysts

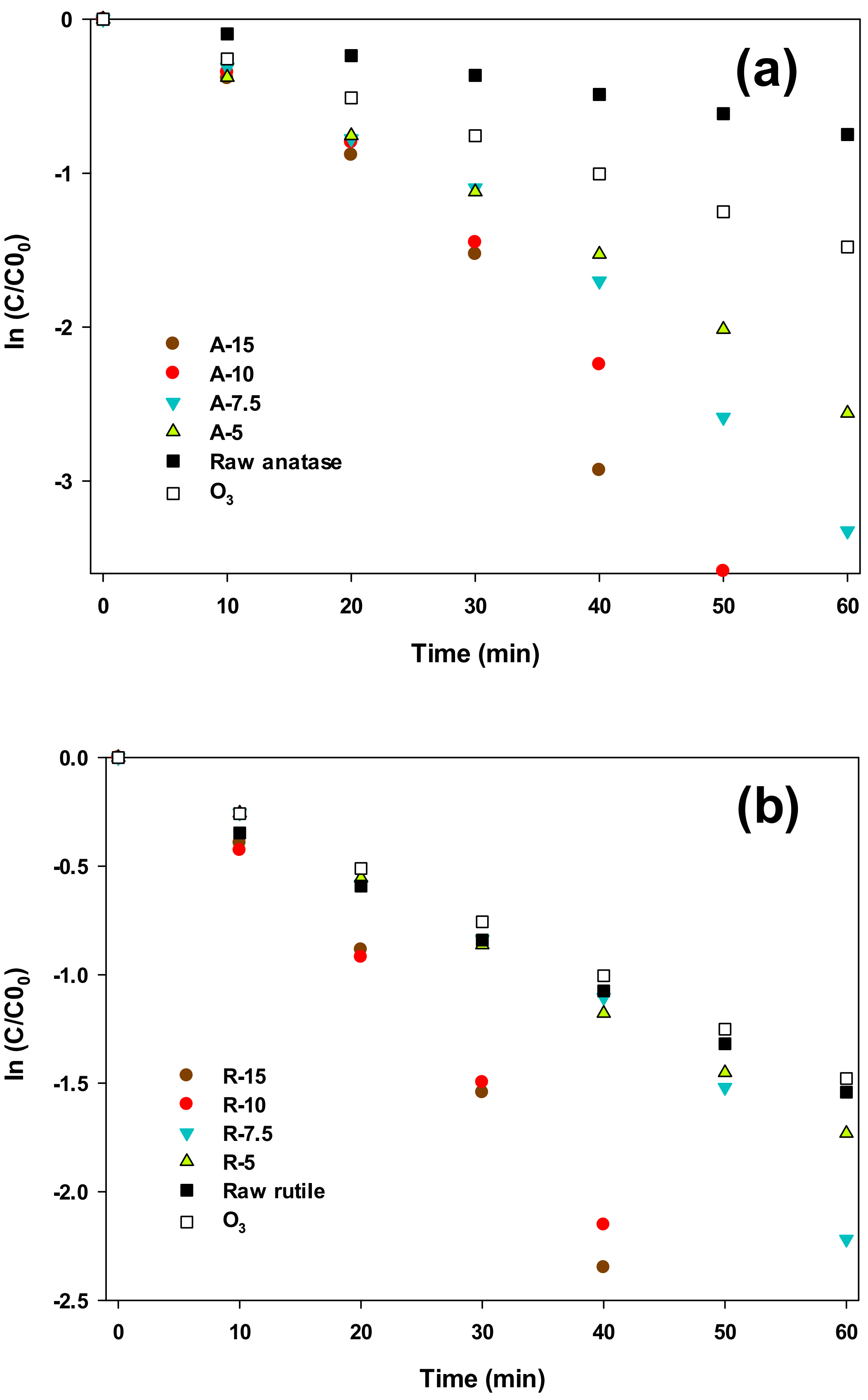

Figure S2 TEM images of catalysts originating from rutile TiO_2 with different concentrations of NaOH in hydrothermal process: (a) and (b) for sample R-5, (c) and (d) for R-7.5, (e) and (f) for R-10, and (g) and (h) for R-15.

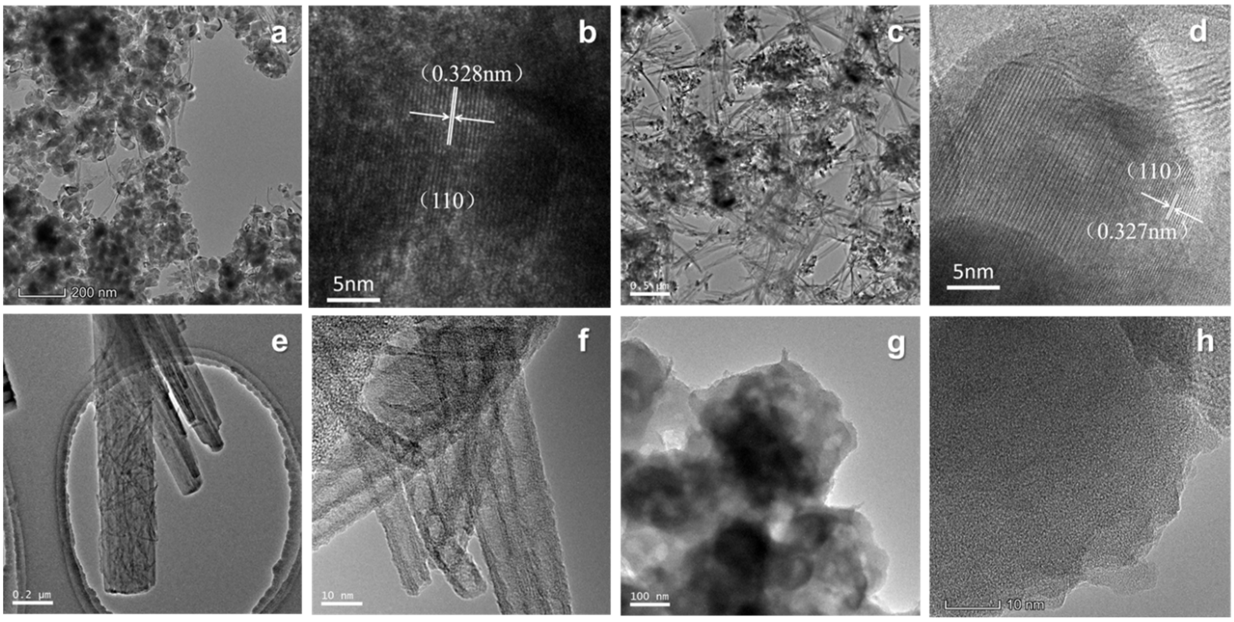

Figure S3 Pore size distribution of titania catalysts originating from rutile

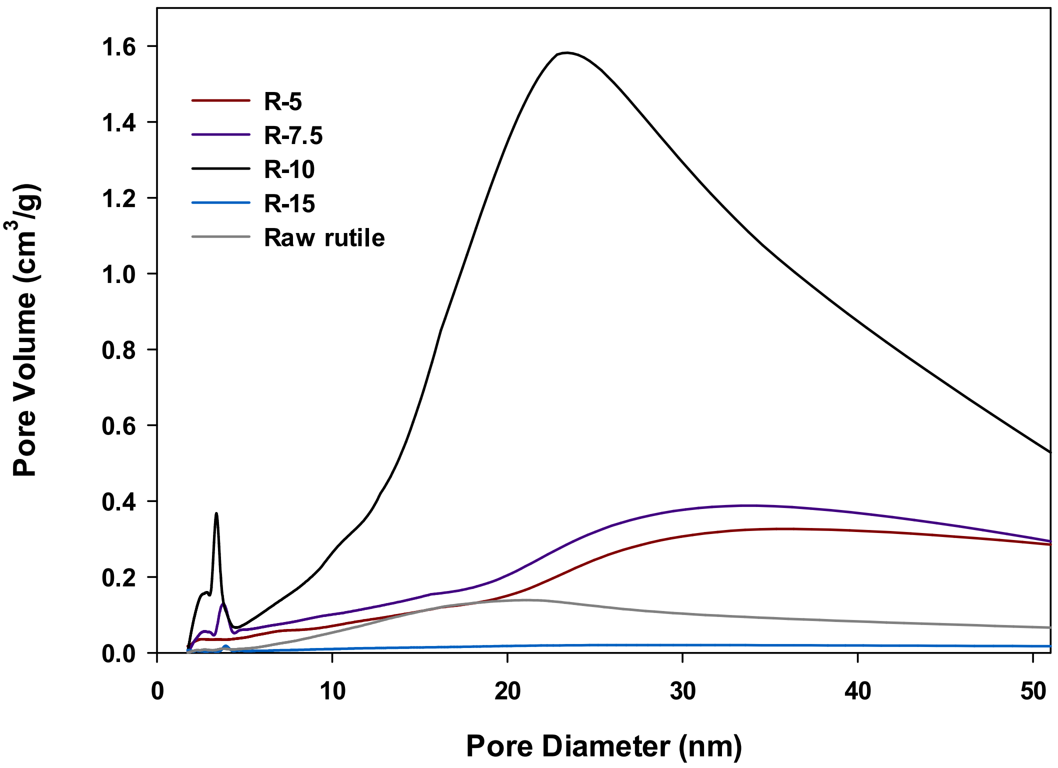

Figure S4 EPR spectra of catalyst R-10 using DMPO as hydroxyl radical trapping agent in water, TEMP as singlet oxygen trapping agent in water, and DMPO as superoxide radical trapping agent in absolute ethanol.

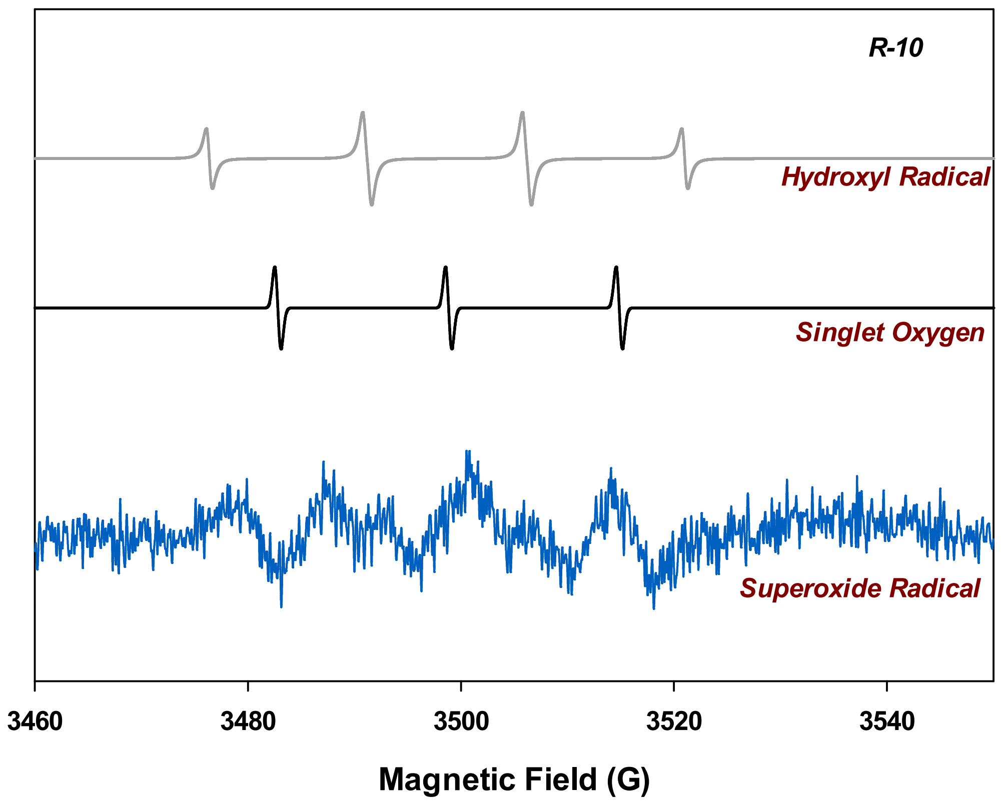

Figure S5 Setup for experiments of catalytic ozonation

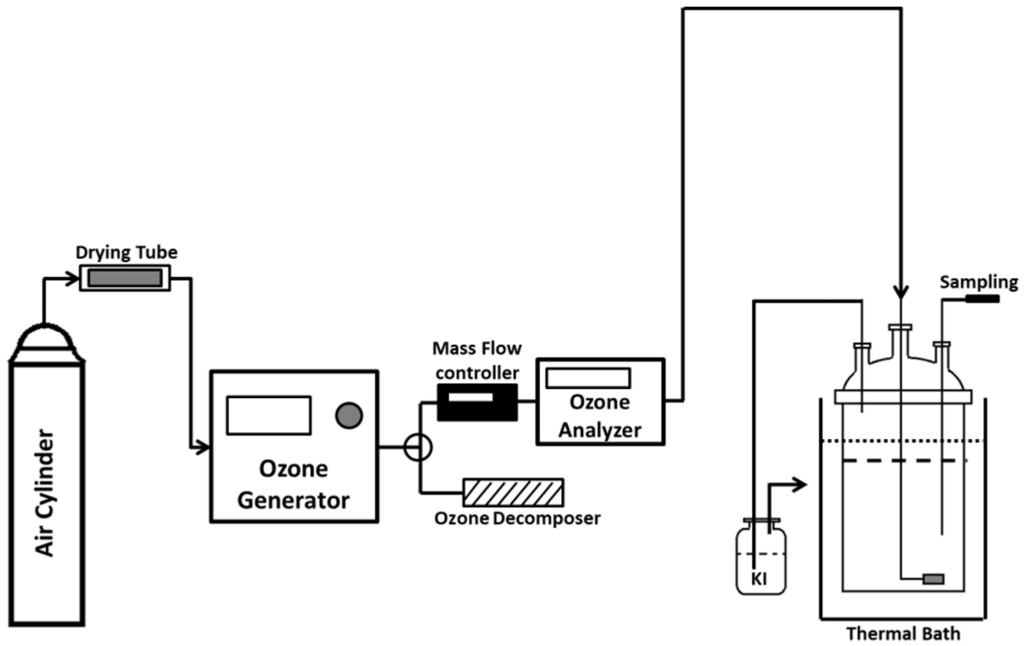

Supplement: Supplementary file 1 [file molecules-27-02706-s001.zip › molecules-1677666-supplementary.pdf]
